# Development of an online teaching platform to improve access to postgraduate pathology training in sub-Saharan Africa

**DOI:** 10.3389/fmed.2024.1390560

**Published:** 2024-05-07

**Authors:** Richard J Byers, Anita J Byers, Chibamba Mumba, Angela Mutuku, Jennifer Singer-Rupp, Michael Wilson, Kenneth Fleming, Shahin Sayed

**Affiliations:** ^1^Division of Cancer Sciences, School of Medical Sciences, Faculty of Biology, Medicine and Health, University of Manchester, Manchester, United Kingdom; ^2^Department of Histopathology, Manchester Royal Infirmary, Manchester University Hospital NHS Foundation Trust, Manchester, United Kingdom; ^3^African Strategies for Advancing Pathology, Denver, CO, United States; ^4^Department of Histopathology, Salford Royal Hospital, Northern Care Alliance NHS Foundation Trust, Salford, United Kingdom; ^5^University of Zambia and University Teaching Hospital, Lusaka, Zambia; ^6^Aga Khan University, Aga Khan University Hospital, Nairobi, Kenya; ^7^Department of Pathology and Laboratory Services, Denver Health and Department of Pathology, University of Colorado School of Medicine, Denver, CO, United States; ^8^Green Templeton College, University of Oxford, Oxford, United Kingdom

**Keywords:** pathology, histopathology, training, digital pathology, online teaching, sub-Saharan Africa

## Abstract

**Background:**

Resource barriers to the provision of accessible training in cancer diagnosis in lower- and middle-income countries (LMICs) limit the potential of African health systems. Long-term provision via teaching visits from senior pathologists and trainee foreign placements is unsustainable due to the prohibitive costs of travel and subsistence. Emerging eLearning methods would allow pathologists to be trained by experts in a cheaper, more efficient, and more scalable way.

**Purpose:**

This study aimed to develop an online teaching platform, starting with hematopathology, for trainee pathologists in sub-Saharan Africa, initially in Nairobi, Kenya, and Lusaka, Zambia.

**Methods:**

Course materials were prepared for both Canvas and the Zoom eLearning platforms using digitally scanned slides of lymph nodes and bone marrow trephines. Initial in-person visits were made to each site to establish trainee rapport and maximize engagement, evaluate different methods and course content, and obtain feedback to develop the project. The knowledge of trainees before and after course completion was used to measure initial effectiveness. Online teaching with the preferred platform is to be continued for 1 year before re-evaluation for long-term effectiveness.

**Results:**

Canvas was selected as the preferred delivery platform as it is freely available and has good functionality to support all required tasks. Face-to-face teaching was considered optimal to establish the initial rapport necessary to maximize subsequent engagement with online teaching. Challenges have included sub-optimal internet speeds and connections and scheduling issues. Weekly online hematopathology teaching sessions using live image capture microscope sessions, Zoom, and Canvas have been delivered to students in Kenya and Zambia, with good attendance and interaction in case discussions.

**Conclusion:**

Our team has successfully designed and delivered an online training program in hematopathology to trainee pathologists in Kenya and Zambia, which has been ongoing for over a year. This project is now being scaled to other sub-Saharan countries and other sub-specialties.

## Introduction

Africa is experiencing an epidemiological transition following the considerable successes of global and national initiatives to improve the detection, prevention, and treatment of infectious diseases. Consequently, as the demographic of sub-Saharan Africa changes and non-communicable diseases become more common, cancer is becoming a leading cause of death (1–4). This brings new challenges of detection and treatment, particularly for cancer treatment (5), with the diagnosis being one of the biggest gaps in the cancer care pathway (6–8), and, despite advances, there remain shortages of specific medical skills, limiting the potential of African health systems (9). Specifically, the average number of pathologists per head of population in Africa is 1/1,000,000, compared to 1/15–20,000 in the US and UK, a 50- to 70-fold difference. Furthermore, pathologists in Africa are focused on a few areas, particularly South Africa and West Africa, and outside these areas, the numbers become very low, with only seven countries having a ratio of less than 1/1,000,000; at least one country has no pathologist. Cancer diagnosis remains technically difficult, and while high-income health systems have developed tools and expertise to meet this challenge, low- and middle-income countries (LMICs) health systems lack key competencies in health workforces, limiting the effectiveness of the care they can offer (10).

However, there have been improvements in the provision of anatomical pathology services in sub-Saharan Africa recently. For example, Zambia has benefitted from external support to increase the number of pathologists in the country from 3 to 20 since 2015 (11). The provision of pathology is more advanced in Kenya, and training in anatomy has been provided at the Aga Khan University Hospital (AKUH) for several years, together with a general pathology training program at the University of Nairobi, although the country still lacks access to specialized molecular diagnostics. Despite these improvements, there remains an ongoing need in both Kenya and Zambia and, by extension, other sub-Saharan African countries to improve expertise in specialist cancer diagnosis, which will require ongoing specialist training (12), and to address the limitations of access to specialized tests and training. The previous specialist training model has relied heavily on external speakers to teach “safaris,” but these are limited in effect due to low frequency, a lack of ongoing education between lecture tours, and cost, both financial and, increasingly, environmental. Moreover, this model of education and training is neither scalable nor sustainable on a long-term basis. Interactive systems, such as the ZOOM Project ECHO, have been successful in facilitating medical training and transferring knowledge between high-income countries and LMICs, enabling long-term sustainable training. We therefore decided to pilot the use of emerging eLearning training methods to train pathologists in cancer diagnosis in a more efficient and scalable manner. As an exemplar, we initially focused on hematopathology diagnostics in Zambia, where we had prior educational experience.

Hematopathology is a particularly challenging area of medicine and is becoming more important as national cancer treatment programs in LMICs mature (13). This study is highly technical, and unfortunately, although medical training for anatomic pathologists has increased in recent years, it is still insufficient to address the need. Zambia, in particular, has seen a recent increase in the number of pathologists, enabling a move to more specialized diagnostics. As such, it is a good model for the maturation and development of high-quality service from a poorly resourced starting point, and thereby a good model of success for other presently poorly resourced LMIC sub-Saharan counties.

We chose Kenya as a second pilot site as an example of a more developed healthcare and training environment to facilitate the development of as strong a base as possible in the region. African Strategies for Advancing Pathology (ASAP) has been involved in cancer diagnostic training for many years and has pioneered training in many countries in Africa using traditional training methods as well as eLearning. Together with the College of Pathologists of East, Central, and Southern Africa (COPECSA) and AKUH, Nairobi, ASAP has organized and delivered several National Cancer Institute (NCI)-funded pathology training workshops, which showed that a blended learning approach yielded the highest educational outcomes (14–16). In view of this, we decided to work with this group to also pilot the hematopathology training program in Kenya.

## Objectives

Two eLearning platforms for training pathologists in hematopathology were compared, one a blend of eLearning (self-directed) teaching modules combined with interactive live sessions delivered remotely through the Zoom platform provided by COPECSA and linked to the platform through ASAP, and the other an eLearning course administered on the Canvas LMS site (self-directed only). The modules were open to senior residents and practicing pathologists at AGUH, Nairobi, Kenya, and the University Teaching Hospital (UTH), Lusaka, Zambia. COPECSA, a regional CME provider for pathology activities for sub-Saharan Africa, was involved in the logistics through the dissemination of the results of the activity to all pathology associations within sub-Saharan Africa.

The objectives of the project were as follows:

1. To develop and evaluate an eLearning course for processing and reporting hematopathology specimens to improve the ability of pathologists in Africa to detect and diagnose leukemia and lymphoma.
2. To develop an online teaching platform in hematopathology for trainee pathologists in Kenya and Zambia initially, with later extension to other sub-Saharan countries.
3. To compare the learning experiences of participants at two separate delivery sites, specifically Kenya and Zambia: a private university hospital, the AGUH, Nairobi, Kenya, and a public university hospital, the UTH, Lusaka, Zambia; both premier sites nationally.
4. To compare Zoom, Canvas, Blackboard, and Moodle as delivery platforms.
5. To assess the subjective experiences of both course organizers and attendees to determine the key criteria in designing and carrying out training on eLearning platforms in the context of sub-Saharan health systems.
6. To use hematopathology as a template to develop a sustainable infrastructure and platform for future eLearning developments in pathology in sub-Saharan Africa, extendable to other sub-specialities.
7. To develop CPD resources to support teaching, including online quizzes related to lectures, PowerPoints, and videos.
8. To engage with trainee residents to ensure the platform and content are appropriate for their needs.
9. To develop an online discussion group in Canvas to support student questions and answers arising from weekly lectures.
10. To provide monthly tutorial sessions.
11. To provide weekly online slide-based teaching.
12. To measure the effectiveness of teaching through pre- and post-course assessments.

## Materials and methods

### Program design, selection, and implementation

The program was designed in collaboration with teaching faculty at the University of Zambia and UTH in Lusaka, and AKUH, Nairobi. The initial teaching program was focused on a comprehensive lecture series covering diagnostic cellular hematopathology, concordant with the needs of the area and the expertise of the teaching team. Subsequent lectures covering other cellular pathology sub-specialities were designed in collaboration with teaching staff at UTH and AKUH and volunteer lecturers.

### Teaching methods and online delivery

**Figure 1:**
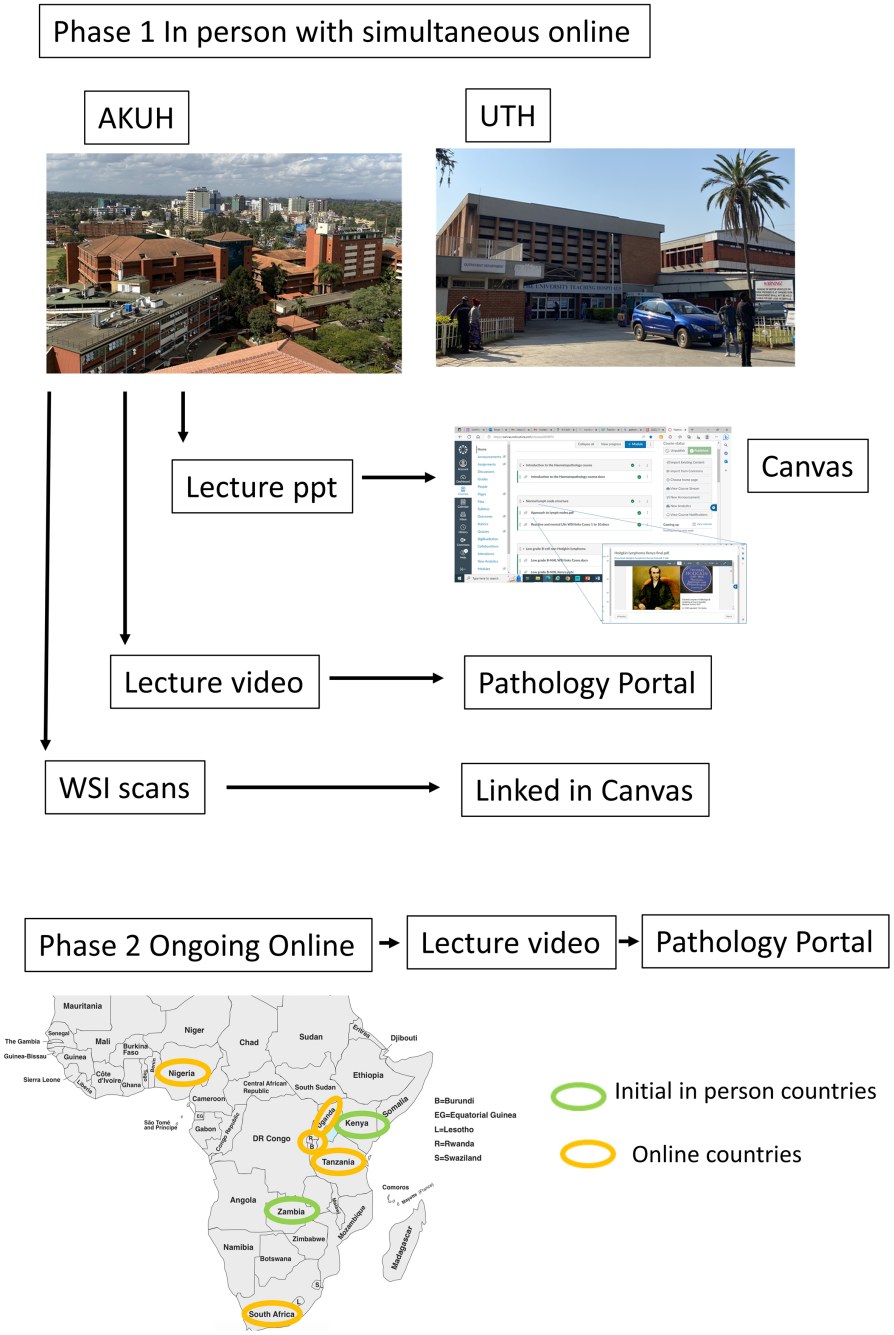
Flow chart illustrating schema of in-person and online lecture delivery, with countries included and organization of teaching material delivery and archiving.

In-person teaching was delivered at the start of the project at UTH and AKUH with simultaneous online attendance via Zoom, with subsequent weekly online seminars. In-person teaching used a combination of didactic teaching and slide seminars using scanned WSIs with a group discussion of cases. All teaching material was uploaded to a Canvas site with links to lecture video recordings, which were uploaded to the Pathology Portal of the Royal College of Pathologists. Weekly online teaching alternated between hematopathology slide seminars using live photomicrograph camera capture of cases and external faculty teaching in other subspecialties, also using a combination of slide seminars and didactic teaching. Group discussion was used to emphasize teaching points and facilitate group Q&A. The program plan is illustrated in Figure 1.

### Monitoring and evaluation

The program was evaluated by student feedback using questionnaires and continual feedback after each teaching session, and the effectiveness of learning was assessed by pre- and post-course assessments.

## Results

### Program delivery

The project delivered a 4-week program of hematopathology teaching in person at the AKUH, Nairobi, Kenya, in May 2022, and a 3-week program at UTH, in September 2022, followed by a second 3-week program at AKUH in May 2023, which also included residents of the University of Nairobi. The lecture material, recorded lectures, Zoom recordings, and whole-slide images (WSIs) used in these teaching sessions formed the basis of an online teaching platform in Canvas for hematopathology, with differentiation into lymphomatous hematopathology focused on anatomical pathology residents and myeloid hematopathology for clinical pathology residents. A comprehensive lecture series covering malignant hematopathology was delivered, specifically including an introduction to lymph nodes, low-grade B-cell NHL, high-grade B-cell NHL, Hodgkin lymphoma, and T-cell NHL, introduction to bone marrow trephines, myeloproliferative neoplasms, myelodysplastic neoplasms, acute leukemia, and myeloma.

The in-person lecture program focused on hematopathology and was developed in conjunction with both the pilot institutions, both of whom had identified hematopathology as a key area, especially given the advances being made in the ability to treat lymphoma and leukemia in both Kenya and Zambia, giving rise to need for more accurate and precise diagnosis to guide targeted therapy. Ongoing lectures cover a wide range of neoplastic and non-neoplastic diseases, aiming to provide an ongoing comprehensive program across all cellular pathology specialties.

### Teaching effectiveness and program development

The program has had a large impact on training in Kenya and Zambia, with approximately 20 pathologists attending in-person teaching at each site. Specifically, at AKU, Nairobi, 20 residents attended each of the two in-person teaching programs, with representation across all years of training (Year 1–5 residents, Year 2–4 residents, Year 3–4 residents, Year 4–5 residents, and 2 fellows, one in Hematology and the other in Onychopathology), while in Lusaka, 23 pathologists attended the in-person teaching (Year 1–5 residents, Year 2–1 resident, Year 3–3 residents, Year 4–4 residents, and 10 consultants). Similarly, the subsequent online teaching has had a large impact on ongoing training across sub-Saharan Africa, with between 20 and 60 pathologists attending weekly from Nigeria, South Africa, Rwanda, Uganda, Tanzania, Kenya, and Zambia, demonstrating wide geographical uptake as planned; lectures given and planned are detailed in Table 1. Pre- and post-course assessments demonstrated improved knowledge of hematopathology.

**Table 1:** Lectures delivered on teaching program.

Hematopathology (delivered online and in person)
Introduction to lymph node pathology
Low-grade B-cell non-Hodgkin lymphoma
High-grade B-cell non-Hodgkin lymphoma
T-cell non-Hodgkin lymphoma
Hodgkin lymphoma
Introduction to bone marrow pathology
Myeloproliferative neoplasms
Myelodysplastic neoplasms
Acute leukemia
Myeloma
Molecular testing in MPN and MDS
Update on 2022 WHO classification of hematological malignancies
Non-hematopathology (delivered online)
Infectious disease 1 Non-HIV virus infections
Infectious disease 2 HIV-direct pathology and prions
Infectious disease 3 Bacteria
Infectious disease 4 Mycobacteria
Infectious disease 5 Fungi
Infectious disease 6 Protozoa
Infectious disease 7 Worms 1
Infectious disease 8 Worms 2
Advances in prostate pathology
What the pathologist needs the clinician to know in breast pathology HER2 low breast cancer
Neuroendocrine lung tumors
Advances in endometrial pathology
Investigation of carcinoma of unknown primary
Liver lesions
Advances in urological pathology
Autopsy pathology
Esophageal tumors

An option appraisal of Canvas, Blackboard, and Moodle as repository platforms for the teaching material identified Canvas as the preferred delivery platform as it is freely available and provides good functionality to support all required tasks (lecture repository, links to Zoom lectures, discussion groups, and quiz section) (Table 2).

**Table 2:** Options appraisal of Canvas, Blackboard, and Moodle as teaching material repository platforms.

Product name	Moodle	Canvas	Blackboard
	https://moodle.com/solutions/moodlecloud/	https://www.lmspulse.com/2021/how-to-install-canvas-lms/	https://help.blackboard.com/Blackboard_App
Basic overview	Open-source learning management solution that is based on a modular design. It enables administrators and teachers to build their own courses using the plug-in. It gives a robust set of functionalities and a collaborative learning environment that helps facilitate both teaching and learning.	Open-source LMS is one of the fastest-growing systems today. It is specifically created for educational institutes – for grades K-12 and higher education. This platform aims to better engage users in their teaching and learning processes. It is ideal for blended learning	Modern, intuitive learning management model that facilitates virtual platforms for learning. It delivers a course management system that has an open architecture. You can combine the system with a student information system and authentication processes.
Free plan	No	Yes	No
Integration capability	Integrates with SIS, Microsoft 365, Google, and Dropbox.	Integrates with SIS and Microsoft 365 by default. However, Dropbox and Google integration require configuration.	Integrates with SIS, Microsoft 365, and Dropbox. However, Google apps integration requires additional apps.
Offline learning	No	Yes	No
Installation method	The cloud version needs no installation but an installer is required for a local onsite version.	The cloud version needs no installation but an installer is required for a local onsite version. Installation can be difficult and requires a Linux server.	The cloud version needs no installation but an installer is required for a local onsite version.
Administrative features	Admin tools enable unparalleled granularity but are less intuitive than other platforms.	Admin tools are easy to use but granularity is lacking.	Admin tools are easy to use but granularity is lacking.
Course development features	Course creation tools are intuitive. The repository system can be hard to navigate, although uploading files and SCORM packages is possible.	Course creation is easiest on Canvas. Uploading files to repositories is easy, and the Course Import Tool enables cross-platform uploads.	Course creation tools are intuitive. Uploading files to repositories is easy, although sharing files across courses requires a paid upgrade.
Assessment method	Assessment tools are extensive and highly accurate, but the Gradebook can be hard to learn.	Assessment tools are easy to learn, and a good level of customization is possible.	The test feature is intuitive, although the Grade Center can be unwieldy.
Communication	Various communication tools (e.g., live chat and discussion forums) and notifications are available	Various communication tools (e.g., live chat and discussion forums) and notifications are available	Various communication tools (e.g., live chat and discussion forums) and notifications are available
Devices and operating system compatibility	Available on all major operating systems, Android and Apple mobile apps available	Available on all major operating systems, Android and Apple mobile apps	Available on all major operating systems, Android and Apple mobile apps

The use of YouTube for ongoing archiving and delivery of lectures delivered both face-to-face and online was tested but not considered to be an optimal solution for the following reasons: (1) the difficulty of post-processing lectures and breaking them into shorter segments for upload, as it is much easier to upload to Google Drive at a low cost; (2) YouTube is open to all, while much of the material delivered is available under permission to use for teaching in the specific context of sub-Saharan Africa but not elsewhere so the open platform is not suitable, while lectures can be made available to the students through a Google drive link which can be made available to all students securely; and (3) YouTube contains advertisements, which many funding organizations will not allow, and also auto-directs to other videos not related to the hematology material. Subsequently, however, since the inception of the project, the Pathology Portal of the Royal College of Pathologists, which has been supported by the National Health Service England Learning Hub, has been made available and selected as the best present option, given it is freely available to international members and provides professionally supported content editing, curation, and maintenance of content covering all aspects of pathology, thereby enhancing the learning opportunities provided by the online teaching program (17).

### Platform appraisal

Initial in-person teaching was considered to be the optimal method for establishing rapport with students necessary to maximize engagement with and benefit from subsequent online teaching. In both Kenya and Zambia, lectures were delivered both in-person and live online at the same time via Zoom, and in both countries, trainee pathologists joined the lectures online, either locally, elsewhere in the country, or from other countries, e.g., Tanzania. WSI images were very effective in teaching and problems of slow internet speed and subsequent image pixelation present in 2022 were not seen in 2023, demonstrating improvement in internet speeds and highlighting the potential for the use of WSI in a sub-Saharan setting.

Feedback collected from the trainees showed high levels of satisfaction with the teaching provided in person, and the uptake of the online material from Canvas has been good. Feedback for online teaching has consistently endorsed the format of alternate hematopathology and external speakers. Hematopathology remains an area of need in sub-Saharan Africa and bi-weekly slide seminar sessions have proved to be particularly useful to improve exposure to advanced diagnostics on an ongoing basis.

### Challenges and solutions

There have been several challenges to overcome in terms of set-up, delivery, and maintenance of the teaching program but all these challenges have been resolved. Internet bandwidth and power supply were initially a problem in both Nairobi and Lusaka but over the period of the pilot (2022–2023), internet availability, reliability, and speed have improved in both locations. While pixelation of downloaded WSIs was common in 2022, with long times for clear image download, this was not a significant issue by 2023. Online delivery has been effective, with the rapid expansion of 4G networks in Kenya and Zambia allowing good Zoom connectivity since the end of 2022. Scheduling across different time zones has been required but has not been difficult as the countries in sub-Saharan Africa are spread over a 4 h time window, thus teaching delivery at midday from the United Kingdom has allowed attendance during working hours from across the region. Similarly, engagement with a large number of pathologists in each of the countries in the region has been facilitated formally by the involvement of COPECSA with the development of the program and informally by wide social network connections across the region.

There is a need to establish internet access levels at other locations in sub-Saharan Africa; this has already been demonstrated as sufficient in Zambia and Kenya but has not been tested in other countries. There is a need to regularly update lecture content as appropriate, and the lecture program is now being extended to other sub-specialist areas, as initially planned. The use of discussion boards in Canvas and of monthly tutorials has not proved necessary given the level of weekly engagement of residents with live online slide-based teaching sessions.

Weekly online slide-based seminars have been delivered via Zoom from Manchester, UK, to residents at AKU, UTH, and UoN using glass slides displayed by live image capture and Zoom, with good attendance (between 20 and 60 per week). Interaction in terms of questions and answers has been effective in these sessions, but they probably would not have worked as well without prior face-to-face delivery. Specifically, the ability for residents to ask questions (Q&A) through the chat function has enabled questions to be collated for answers, while the interactive nature of Zoom for slide seminar delivery has facilitated active learning through teacher question prompts and instant answer feedback to clarify diagnostic points. The weekly teaching and associated Q&A using the Zoom chat function have obviated the need for separate discussion groups and tutorials as initially planned in objectives 9 and 10, delivering a more streamlined, time-efficient learning environment.

## Conclusion

The project has successfully delivered an online training program in hematopathology to trainees in Kenya and Zambia, ongoing now for over a year. This project can now be scaled to other sub-Saharan countries and to other sub-specialities.

## Discussion and future work

The pilot project has demonstrated the feasibility of online teaching in sub-Saharan Africa and is now being extended to other countries beyond the “founder” countries of Zambia and Kenya, most recently to Tanzania, Rwanda, Nigeria, South Africa, and Uganda, and sub-specialities other than hematopathology. The uptake of the program has been good, and in Kenya and Zambia approximately 40 and 20 residents, respectively, have participated in both initial face-to-face and ongoing online teaching over the past 18 months, while between 20 and 60 pathologists attend ongoing weekly online teaching. As such, the teaching provided has become a fixed part of the program of resident teaching in both Kenya and Zambia and is being extended to other sub-Saharan countries. An increasingly large faculty is being recruited, many of whom are keen to contribute to teaching abroad within an established and active platform but lacked the resources to establish the platform and recruit a cadre of learners. To further establish the program and maximize the value of participation in it to residents and consultant pathologists, a key next step is the conversion of the teaching undertaken into CDP modules to accompany the lectures. This will best be done in collaboration with the College of Pathologists of East, Central, and Southern Africa (COPECSA) countries to develop an accreditation program for online teaching across the region, specifically in Tanzania, Uganda, Kenya, Burundi, Zambia, Rwanda, Madagascar, Botswana, and Zimbabwe. Additionally, given the large amount of online teaching that is now being provided, there is a need for a website collating, in live-diary format, online teaching events open to pathologists in sub-Saharan Africa in order to maximize dissemination of both the teaching provided under the proposed program and that provided by others. COPECSA would be the natural partner for the development of this, possibly in conjunction with a diagnostic commercial partner.

ASAP has previously provided training courses for cancer staging and has developed an eLearning course on this topic (16), giving the experience to be used as a template for planned online teaching in hematopathology, while the lessons learned through application in hematopathology are transferable to other subspecialties. The recent pandemic has limited travel, especially in sub-Saharan Africa, emphasizing the need for and driving the development of reliable virtual conferencing in Africa, which now allows a newer type of training to be envisioned. This type of model has significant advantages, especially for LMICs, for which travel costs can be prohibitive. Interactive systems, such as the ZOOM Project ECHO, have been successful in facilitating medical training and the transfer of knowledge between high-income countries and LMICs (18). The pilot project described builds upon earlier experience to assess and develop the best of these new technologies to establish a platform for future pathology training to increase capacity in African health systems. In recent years, advances in telecommunications and improved connectivity in LMICs have combined to allow a number of exciting new teaching methods to emerge. Project Zoom ECHO is a knowledge-sharing network that links expert medical teams with primary care clinicians in rural and urban underserved locations. This technology offers a chance to conduct case-based training remotely, thus benefiting from the immediate and interactive nature of this approach but being vastly more efficient in terms of cost, time, and other valuable resources than traditional in-person teaching. If this approach proves effective, it will enable a teaching platform that saves money, carbon dioxide, and clinicians’ time, while vastly increasing accessibility to education.

To date, teaching has been focused on hematopathology, as stated in the initial pilot program. This was an area of need requested by pathologists in Kenya and Zambia, and fortuitously, the specialist area of the program lead, Dr. Byers. It was the stated aim of the pilot to extend the program into other areas, and this has now commenced, with planned lectures on area-relevant topics, including HIV-related lymphoma, breast cancer, prostate cancer, and renal pathology, and the recruitment of lecturers from sub-Saharan Africa to provide geographical relevance. It is also recognized that as the program grows, it will be necessary to extend the number of trainers to facilitate dissemination of the program, ensure continuity, and maximize local relevance. COPECSA and contacts in South Africa will be used to identify a cadre of pathologists in each of the COPECSA countries who will be trained to support the program locally and, in time, more generally. The aim is to have a senior training mentor and junior trainer in each country; the senior trainer will provide continuity, and junior members will have strong collaboration with peers. We have already identified trainers for this role in Kenya and Zambia.

Finally, and most importantly, despite improvements in prevention and detection, and in medical, surgical, and pathology services in sub-Saharan Africa, death rates from cancer continue to rise, largely due to changing demographics and the adoption of Western lifestyles. Improved outcomes for these emerging health problems will only be effectively addressed by an integrated end-to-end (E2E) healthcare program that includes and optimizes all stages of the patient journey, from prevention and detection to treatment (19). Better access to diagnostics is a key part of this journey, and the vital link between diagnostics and medical/surgical outcomes has recently been recognized in the WHO resolution on diagnostics (20). This, in turn, requires both (1) better access to and number of pathologists and (2) an improved quality of diagnosis. The extension of the geographical spread and content of the program will therefore not achieve maximal benefit except as part of an E2E program. Consequently, in order to ensure the relevance of the program to a local E2E approach to improving cancer outcomes, it will be necessary to include oncologists and surgeons to identify their concerns and ensure they are been addressed. The expansion of the teaching is planned based on such discussions, and the lead author (RJB) is working with colleagues at the University of Manchester who are delivering an E2E project to improve outcomes in esophageal cancer, lessons from which will facilitate the integration of improved diagnostics in improved patient pathways for other cancers (21–23).

The pilot project has successfully demonstrated the value and scalability of an e-learning platform for pathology residents in sub-Saharan Africa and (24, 25) is now ready for expansion in content and geographical scope beyond the founding countries of Zambia and Kenya. It will be necessary to include it in an E2E approach to ensure maximal benefit in improving cancer outcomes.

## Data availability statement

The original contributions presented in the study are included in the article/supplementary material, further inquiries can be directed to the corresponding author.

## Author contributions

RB: Writing – review & editing, Writing – original draft, Supervision, Project administration, Methodology, Investigation, Funding acquisition, Data curation, Conceptualization. AB: Writing – review & editing, Writing – original draft, Project administration, Data curation, Conceptualization. CM: Writing – review & editing, Writing – original draft, Supervision, Resources, Project administration, Methodology, Funding acquisition, Conceptualization. AM: Writing – review & editing, Resources, Supervision, Project administration, Funding acquisition. JS-R: Writing – review & editing, Resources, Project administration, Funding acquisition. MW: Writing – review & editing, Writing – original draft, Supervision, Project administration, Methodology, Funding acquisition, Conceptualization. KF: Writing – review & editing, Writing – original draft, Supervision, Project administration, Methodology, Funding acquisition, Conceptualization. SS: Writing – review & editing, Writing – original draft, Supervision, Resources, Project administration, Methodology, Funding acquisition, Conceptualization.

## References

[1] Shehu MN Adamu UG Ojji DB Ogah OS Sani MU. The pandemic of coronary artery disease in the sub-Saharan Africa: what clinicians need to know. Curr Atheroscler Rep. (2023) 25:571–8. doi: 10.1007/s11883-023-01136-9, PMID: 37606811

[2] Mudie K Jin MM Kendall L Addo J dos-Santos-Silva I Quint J. Non-communicable diseases in sub-Saharan Africa: a scoping review of large cohort studies. J Glob Health. (2019) 9:20409. doi: 10.7189/jogh.09.020409, PMID: 31448113 PMC6684871

[3] Ngwa W Addai BW Adewole I Ainsworth V Alaro J Alatise OI. Cancer in sub-Saharan Africa: a lancet oncology commission. Lancet Oncol. (2022) 23:e251–312. doi: 10.1016/S1470-2045(21)00720-8, PMID: 35550267 PMC9393090

[4] Cohee LM Opondo C Clarke SE Halliday KE Cano J Shipper AG. Preventive malaria treatment among school-aged children in sub-Saharan Africa: a systematic review and meta-analyses. Lancet Glob Health. (2020) 8:e1499–511. doi: 10.1016/S2214-109X(20)30325-9, PMID: 33222799 PMC7721819

[5] Omotoso O Teibo JO Atiba FA Oladimeji T Paimo OK Ataya FS. Addressing cancer care inequities in sub-Saharan Africa: current challenges and proposed solutions. Int J Equity Health. (2023) 22:189. doi: 10.1186/s12939-023-01962-y, PMID: 37697315 PMC10496173

[6] Bray F Parkin DM Gnangnon F Tshisimogo G Peko JF Adoubi I. Cancer in sub-Saharan Africa in 2020: a review of current estimates of the national burden, data gaps, and future needs. Lancet Oncol. (2022) 23:719–28. doi: 10.1016/S1470-2045(22)00270-4, PMID: 35550275

[7] Jedy-Agba E McCormack V Adebamowo C Dos-Santos-Silva I. Stage at diagnosis of breast cancer in sub-Saharan Africa: a systematic review and meta-analysis. Lancet Glob Health. (2016) 4:e923–35. doi: 10.1016/S2214-109X(16)30259-5, PMID: 27855871 PMC5708541

[8] Akuoko CP Armah E Sarpong T Quansah DY Amankwaa I Boateng D. Barriers to early presentation and diagnosis of breast cancer among African women living in sub-Saharan Africa. PLoS One. (2017) 12:e0171024. doi: 10.1371/journal.pone.0171024, PMID: 28192444 PMC5305236

[9] Fagan JJ. Workforce considerations, training, and diseases in Africa. Otolaryngol Clin N Am. (2018) 51:643–9. doi: 10.1016/j.otc.2018.01.009 29496257

[10] Gray IP Carter JY. An evaluation of clinical laboratory services in sub-Saharan Africa: ex Africa semper aliquid novi? Clin Chim Acta. (1997) 267:103–28. doi: 10.1016/S0009-8981(97)00180-0 9469247

[11] Mudenda V Malyangu E Sayed S Fleming K. Addressing the shortage of pathologists in Africa: creation of a MMed Programme in pathology in Zambia. Afr J Lab Med. (2020) 9:974. doi: 10.4102/ajlm.v9i1.974, PMID: 32537426 PMC7276345

[12] The Royal College of Pathologists. Rcpath. Available at: https://www.rcpath.org/international/projects/labskills-africa.htmlath.org (Accessed November 30, 2023)

[13] Okello CD Niyonzima N Ferraresso M Kadhumbula S Ddungu H Tarlock K. Haematological malignancies in sub-Saharan Africa: East Africa as an example for improving care. Lancet Haematol. (2021) 8:e756–69. doi: 10.1016/S2352-3026(21)00198-8, PMID: 34481552

[14] Nelson AM Hale M Diomande MIJ Eichbaum Q Iliyasu Y Kalengayi RM. Training the next generation of African pathologists. Clin Lab Med. (2018) 38:37–51. doi: 10.1016/j.cll.2017.10.004, PMID: 29412884

[15] Wilson ML Ayers S Berney D Eslan A Guarner J Lester S. Improving anatomic pathology in sub-Saharan Africa to support Cancer care. Am J Clin Pathol. (2018) 149:310–5. doi: 10.1093/ajcp/aqx158, PMID: 29471457 PMC6144773

[16] Sayed S Lester SC Wilson M Berney D Masia R Moloo Z. Creation and pilot testing of cases for case-based learning: a pedagogical approach for pathology cancer diagnosis. Afr J Lab Med. (2017) 6:637. doi: 10.4102/ajlm.v6i1.637, PMID: 29147646 PMC5680453

[17] Pathology portal. The Royal College of Pathologists. Available at: https://www.rcpath.org/profession/pathology-portal.html (Accessed March 14, 2024)

[18] Dreizler L Wanjiku GW. Tele-ECHO for point-of-care ultrasound in rural Kenya: a feasibility study. R I Med J. (2019) 102:28–31. PMID: 31480816

[19] Mutebi M Anderson BO Duggan C Adebamowo C Agarwal G Ali Z. Breast cancer treatment: a phased approach to implementation. Cancer. (2020) 126:2365–78. doi: 10.1002/cncr.32910, PMID: 32348571

[20] WHO. (2023). Strengthening diagnostics capacity, seventy-sixth world health assembly. Available at: https://apps.who.int/gb/ebwha/pdf_files/WHA76/A76_R5-en.pdfo.int (Accessed November 30, 2023)

[21] NIHR (2022). Global Health research Group for Improving Oesophageal Cancer Survival in Kenya: the hub and spoke model. Available at: https://fundingawards.nihr.ac.uk/award/NIHR133382 (Accessed March 14, 2024)

[22] Fleming KA Horton S Wilson ML Atun R DeStigter K Flanigan J. The lancet commission on diagnostics: transforming access to diagnostics. Lancet. (2021) 398:1997–2050. doi: 10.1016/S0140-6736(21)00673-5, PMID: 34626542 PMC8494468

[23] Sayed S Cherniak W Lawler M Tan SY El Sadr W Wolf N. Improving pathology and laboratory medicine in low-income and middle-income countries: roadmap to solutions. Lancet. (2018) 391:1939–52. doi: 10.1016/S0140-6736(18)30459-8, PMID: 29550027

[24] Ayad E Yagi Y. Virtual microscopy beyond the pyramids, applications of WSI in Cairo University for E-education & telepathology. Anal Cell Pathol (Amst). (2012) 35:93–5. doi: 10.1155/2012/124076, PMID: 22297472 PMC4605605

[25] Domgue JF Dille I Fry L Mafoma R Bouchard C Ngom D. Enhancing cervical and breast cancer training in Africa with e-learning. Lancet Glob Health. (2023) 11:e28–9. doi: 10.1016/S2214-109X(22)00499-5, PMID: 36521948

